# Iron Solubility and Uptake in Fava Bean and Maize as a Function of Iron Chelates under Alkaline Hydroponic Conditions

**DOI:** 10.1021/acs.jafc.5c08914

**Published:** 2025-10-22

**Authors:** Muhammad Faizan Ilyas, Muhammad Imran, Asif Naeem, Arjen M. Reichwein, Karl Hermann Mühling

**Affiliations:** † Institute of Plant Nutrition and Soil Science, 9179 Kiel University, Hermann-Rodewald-Strasse 2, 24118 Kiel, Germany; ‡ Nouryon Research, Development and Innovation (RD & I) Center, Zutphenseweg 10, 7418 AJ Deventer, The Netherlands

**Keywords:** chelated micronutrients, nonchelated micronutrients, alkalinity, iron deficiency, nutrient precipitation, Fe-EDTA, Fe-DTPA, Fe-HBED

## Abstract

Iron-EDTA becomes unstable above pH 6.5, resulting in competition among metal ions (Fe, Cu, Zn, and Mn) for EDTA and reduced Fe availability. This study examined nutrient precipitation dynamics and interactions between chelated and sulfate forms of Zn, Mn, and Cu with Fe-EDTA in fava bean and maize under increasing alkalinity (0, 5, and 15 mM NaHCO_3_). In fava bean, chelated micronutrients significantly increased shoot Fe, P, and Zn concentrations and improved metabolic activity and oxidative stress tolerance. Maize was more sensitive to alkalinity but showed similar nutrient responses at 5 mM NaHCO_3_. Speciation modeling (MINEQL+5.0) indicated Fe displacement from Fe-EDTA at high pH, forming insoluble FePO_4_ and Fe­(OH)_3_. Incubation studies demonstrated that Fe-HBED maintained higher Fe, Ca, and P solubility than Fe-EDTA and Fe-DTPA under alkaline conditions. SEM-EDX analysis linked stable Fe chelates to lower P in precipitates. Results highlight Fe-HBED’s effectiveness in ensuring Fe and P availability under alkalinity.

## Graphical abstract

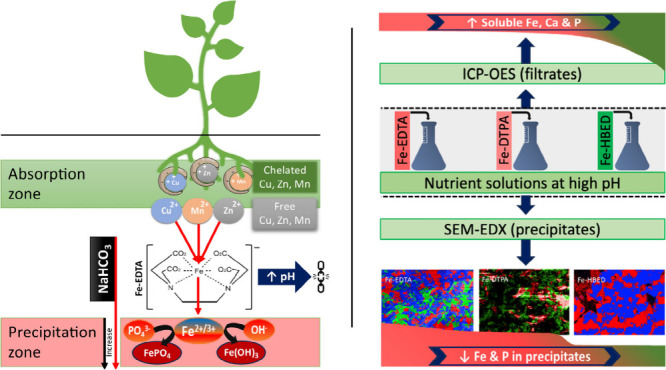

## 1 Introduction

Soil salinity is a major abiotic stress that adversely affects crop growth and productivity.^1^ Alarmingly, an estimated 1.5 million ha of arable land are rendered unsuitable for cultivation each year due to salinization. If this trend continues, it is predicted that up to 50% of the world’s cultivable land could be lost by 2050.^2^ In many natural environments, salinity is often accompanied by alkalinity, which is mainly constituted of bicarbonate (HCO_3_^–^) and carbonate (CO_3_^2–^) salts.^3^ The coexistence of these two stresses compounds the challenge as the excess amounts of HCO_3_^–^ and CO_3_^2–^ increase the pH of rhizosphere.^4,5^ Iron availability to plants is often limited in calcareous and alkaline growth conditions, which occupy approximately 30% of the world’s cultivated land.^6^ The primary reason for Fe deficiency in these soils is its low solubility under alkaline pH, where ferric ions (Fe^3+^) rapidly precipitate as insoluble hydroxides and phosphates, resulting in impaired physiological functions and Fe deficiency-induced chlorosis in plants.^7,8^ It has been estimated that the bioavailability of Fe reduces 1000 times per unit increase in pH.^9^ Mahmoudi et al. found that the effects of bicarbonate-induced Fe deficiency on plant physiology were more pronounced than those of direct Fe deficiency.^10^

Plants growing under alkaline stress often exhibit reduced biomass production, impaired nutrient uptake, and increased production of reactive oxygen species, leading to oxidative damage.^11^ In response, plants adopt several adaptive mechanisms, including enhanced synthesis of organic acids, activation of antioxidant defense systems, and modulation of root architecture to improve acquisition of nutrients, particularly of P and Fe.^12–14^ Moreover, the Fe acquisition mechanism varies between nongraminaceous and graminaceous plants. The nongraminaceous plants utilize the reduction-based strategy (Strategy I), while the graminaceous plants employ the chelation-based strategy (Strategy II) to facilitate Fe uptake from rhizosphere.^15,16^ To combat Fe deficiency under alkaline environments, Fe chelates are widely used, as chelating agents can maintain Fe in soluble and plant-available forms even at higher pH levels.^17–20^ Among these, Fe-EDTA and Fe-DTPA have been extensively applied due to their relative cost-effectiveness and moderate stability. However, their practical efficiency declines with an increase in soil alkalinity. Fe-EDTA has been reported to lose stability above pH 6.5, while Fe-DTPA shows reduced stability above pH 7.0, limiting their utility in highly alkaline soils.^21^ This suggests that a more stable source of Fe is indispensable to ensure adequate Fe uptake by plants at higher pH values. Recently, Fe-HBED has emerged as a superior alternative due to its exceptionally high stability constant (log *K* ≈ 39) and greater resistance to competitive displacement by other cations. Its structural features, such as strong binding affinity and steric hindrance, make it particularly suitable for maintaining Fe solubility in alkaline conditions.^22,23^

The application of different Fe chelates could influence not only Fe availability but also affect the uptake and homeostasis of other essential nutrients such as phosphorus (P), copper (Cu), manganese (Mn), zinc (Zn), and calcium (Ca) within the rhizosphere.^24–26^ Sodium bicarbonate reduces the plant available Fe, Zn, and Mn which ultimately reduces root ferric reduction capacity and expression of the genes responsible for Fe acquisition.^27,28^ Studies highlighted the need of using chelated Zn, Mn, and Cu along-with Fe.^29,30^ However, the mechanism by which free cations displace Fe from Fe-EDTA, leading to Fe deficiency in plants under NaHCO_3_-induced alkalinity, is poorly understood. The intricate nutrient interactions under alkaline conditions can intensify precipitation reactions in the rhizosphere and ultimately cause ion imbalances in the plants. Calcium ions, in particular, could play a significant role in destabilizing intermediate-strength Fe chelates (such as Fe-EDTA) by promoting precipitation reactions of Fe and Ca. Despite a few investigations into Fe–P interactions, their broader implications for the overall nutrient uptake system in plants growing under alkaline stress have remained poorly characterized. Notably, while the individual effects of micronutrient chelates and their competition with Fe uptake are relatively well documented, their collective impact when applied in combination is less understood, especially under high alkalinity conditions.

Given these gaps, a hydroponics experiment was conducted to evaluate Fe uptake by fava bean and maize plants when Fe-EDTA was supplied in combination with either EDTA-chelated forms of other micronutrients (Cu, Zn, Mn) or their corresponding sulfate salts. This experimental setup aimed to assess whether the chemical form of accompanying micronutrients influences Fe uptake and plant physiological responses under alkaline conditions. Iron chelates are effective for Strategy I plants but less efficient for Strategy II plants, as the chelates can compete with phytosiderophores in Strategy II plants,^18^ whereas, sodium bicarbonate induced high pH adversely affect the efficacy of Strategy I.^8,31^ Fava bean and maize were chosen for this study to uncover the treatments’ effects on nongraminaceous and graminaceous plants since these have different Fe uptake mechanisms. The nutrient speciation in the nutrient solutions used for the hydroponics study was also predicted. Second, an incubation experiment was conducted to examine the soluble nutrient concentrations and nutrient precipitation patterns under varying levels of NaHCO_3_ alkalinity over time using Fe-EDTA, Fe-DTPA, and Fe-HBED as Fe sources. By integrating plant physiological assessments with nutrient solution chemistry and precipitation dynamics, this research aims to bridge the knowledge gap between plant nutritional responses and the underlying chemical processes governing nutrient availability in alkaline environments.

It was hypothesized that (1) Free Cu, Zn, and Mn ions in the nutrient solution cause Fe deficiency in plants by replacing Fe from Fe-EDTA, and (2) Fe-HBED maintains higher soluble Fe and P concentrations in alkaline nutrient solution by reducing their precipitation.

## 2 Materials and Methods

### 2.1 Experiment with Fava Bean

#### 2.1.1 Selection of Sodium Bicarbonate Alkalinity Levels

A preliminary hydroponic experiment was conducted to identify suitable levels of NaHCO_3_ alkalinity while considering the growth of fava bean plants and the stability of EDTA. For this purpose, fava bean plants were grown in nutrient solution containing six levels of NaHCO_3_ viz. 0, 1, 2, 5, 15, and 30 mM, with the recorded corresponding nutrient solution pH values of 5.2, 7.0, 7.3, 7.9, 8.4 and 8.6, respectively. Hereafter, the NaHCO_3_ levels are called as “alkalinity levels”. The growth of fava bean plants remained unaffected up to 5 mM NaHCO_3_ alkalinity, whereas 15 and 30 mM NaHCO_3_ had drastic adverse effects on the plant growth. The pH of the nutrient solution containing 15 mM NaHCO_3_ was slightly above the threshold at which the Fe-EDTA complex dissociates (pH ≈ 8.2), and plants still produced sufficient biomass. On the other hand, the pH of nutrient solution containing 30 mM NaHCO_3_ was far above the pH level at which Fe-EDTA complex dissociates, and plants produced very low biomass due to severely damaged roots at the early growth stage. Thus, 0, 5, and 15 mM NaHCO_3_ levels were selected for further studies.

#### 2.1.2 Plant Cultivation and Treatment Application

A hydroponic experiment was conducted in the growth chamber of the Institute of Plant Nutrition and Soil Science at Kiel University. Day/night durations were 14 and 10 h, temperatures were 20 and 15 °C, relative humidities were 50% and 60%, respectively, and photosynthetic photon flux density was 200 μmol m^–2^ s^–1^. The seeds of the fava bean (*Vicia faba* var. Fuego), presoaked in continuously aerated 0.5 mM CaSO_4_ solution for 24 h, were germinated using sandwich blots. The nine-day-old homogeneous seedlings were transferred to 2.5-L pots containing continuously aerated nutrient solution. The experiment was comprised of six treatments with four replicates. The three sodium bicarbonate alkalinity levels (mM NaHCO_3_) developed in nutrient solution were 0 (pH 5.2), 5 (pH 7.9), and 15 (pH 8.4). Fe-EDTA was used as Fe source, in combination with either EDTA chelates or sulfates of Cu, Zn, and Mn. Na-free micronutrients–EDTA chelates were used in the study to avoid any additional Na input into the nutrient solution. The nutrient solution was replaced twice a week. The composition of the nutrient solution was as follows: macronutrients (mM): Ca (NO_3_)_2_ = 2, (NH_4_)_2_SO_4_ = 0.35, K_2_SO_4_ = 0.5, KH_2_PO_4_ = 0.2, CaCl_2_ = 1.3, MgSO_4_ = 0.5, KCl = 1.0; micronutrients (μM): Fe-EDTA = 200, H_3_BO_3_ = 5, MnSO_4_/Mn-EDTA= 2, ZnSO_4_/Zn-EDTA = 0.5, CuSO_4_/Cu-EDTA = 0.3, and (NH_4_)_2_Mo_7_O_24_ = 0.01. The equal concentrations of Cu, Zn, and Mn in chelated and sulfated micronutrient stock solutions were confirmed by analysis with AAS (S4 AA System, Sr. No, GE711932, Thermo-Electron-Corporation, China).

#### 2.1.3 Harvesting and Nutrient Analysis

The nondestructive measurement of leaf chlorophyll contents was carried out on youngest fully expanded leaves (YFEL) after 4 weeks of transplanting using a SPAD meter (SPAD-502, Minolta Co., Ltd. Japan). Plants were harvested after 4 weeks of growth in nutrient solution. The shoots and roots of fava bean plants were separated, washed thoroughly with running deionized water, and oven-dried at 65 °C until constant weight. After oven drying, the dry weights of the samples were recorded and ground to fine powder, and 0.2 g of sample was digested in 10 mL of concentrated (69%) nitric acid using microwave digestion system (MARS 6, CEM Matthews, NC, USA). The digests were diluted to 100 mL, and the phosphorus and micronutrient concentrations in the digests were determined by ICP-OES (Agilent 5800, Agilent Technologies Inc., 2021, USA).

#### 2.1.4 Measurement of Anions and Organic Acids

Water-soluble anions and organic acids were extracted from the shoot and root of fava bean by hot water. Briefly, 1.5 mL of Milli-Q H_2_O was added in 2 mL reaction tubes containing 20 mg of oven-dried finely ground plant sample. The tubes were properly capped and immersed in boiling water for 5 min, and afterward immediately placed in an ice bath for 30 min. The samples were then centrifuged at 4 °C and 12000 rpm for 10 min. The supernatants so obtained were purified by adding chloroform and centrifuged again at 4 °C and 12000 rpm for 5 min. The supernatants obtained were passed through strata C-18 columns (Phenomenex, Torrance, CA, USA), and the anions and organic acids were measured in them by IC (IC-5000 Capillary Reagent-Free IC System, Thermo Scientific, USA).

#### 2.1.5 Determination of H_2_O_2_ and MDA Concentration

The leaf H_2_O_2_ and MDA concentrations in leaves were measured following Velikova et al.^32^ Briefly, 0.5 g of fresh sample was homogenized in 5 mL of 0.1% trichloroacetic acid (w/v) at 0 °C. For H_2_O_2_, the homogenate was centrifuged at 12,000 × *g* for 15 min, 0.5 mL of supernatant was transferred to 2 mL tubes, and 0.5 mL of 10 mM KP buffer (pH 7) and 1 mL of 1 M KI were added into it. The absorbance was measured at 390 nm. A series of H_2_O_2_ standards were analyzed to create a standard curve for calculating H_2_O_2_ concentrations of the unknown samples.

For MDA, the homogenate was centrifuged at 10,000*g* for 20 min at 4 °C. The reaction mixture, containing 0.5 mL of supernatant and 1 mL of 0.5% TBA in 20% TCA in 2 mL tubes, was incubated in boiling water for 30 min and immediately transferred to an ice bath. The tubes were centrifuged at 10,000 × *g* for 5 min, and absorbance was measured at 532 and 600 nm by spectrophotometer. The MDA concentration was calculated using an extinction coefficient of 155 mM^–1^ cm^–1^.

### 2.2 Experiment with Maize

To determine the effect of chelated and nonchelated micronutrients on nutrient uptake, maize plants were grown hydroponically in the green house of the Institute of Plant Nutrition and Soil Science at Kiel University. Day and night durations were 14 and 10 h, temperatures were 23 ± 2 °C and 15 ± 2 °C, relative humidity levels were 50% and 60%, respectively, and photosynthetic photon flux density was 250 μmol m^–2^ s^–1^. The treatments and plant cultivation methods were similar to those for the fava bean experiment. The plants were harvested after 4 weeks of growth in 5 L pots containing continuously aerated nutrient solution, and the nutrient concentrations in shoot and root were determined by ICP-OES following digestion in concentrated (69%) nitric acid as described in section 2.1.3.

### 2.3 Modeling Nutrients’ Precipitation in Nutrient Solution under Varyon Alkalinity Levels

Precipitation of different nutrients in the nutrient solution at different alkalinity levels was calculated by the Chemical Equilibrium Modeling System MINEQL+ 5.0. The concentrations used for speciation calculations in MINEQL+ were based on the nutrient solution composition. The different Na concentrations viz 0, 5, and 15 mM reflect the addition of, respectively 0, 5, and 15 mM NaHCO_3_ in nutrient solution. The different K, SO_4_^2–^ and EDTA concentrations reflect, respectively, nonfully chelated micronutrients (EDTA salt of Fe, while sulfate salts of Cu, Mn, and Zn) and fully chelated micronutrients (EDTA chelates of Fe, Cu, Mn, and Zn). In fully chelated micronutrients, the concentration of EDTA and K was 2.8 and 6.0 μM higher, respectively, while that of SO_4_^2–^ was 3.0 μM lower compared to partially chelated micronutrients. The speciation calculations were run from pH 4 to pH 10 using the calculated ionic strength.

### 2.4 Time-Dependent Relative Iron (Fe) Solubility in the Nutrient Solution of Variable Alkalinity Containing Different Fe Chelates

An incubation experiment was conducted to evaluate time-dependent soluble Fe in the nutrient solution of variable alkalinity using different Fe-chelates viz. Fe-EDTA, Fe-DTPA, and Fe-HBED. The same composition of nutrient solution and NaHCO_3_ levels were used as described in section 2.1.2. Nutrient solutions prepared by using different Fe-chelates with variable levels of NaHCO_3_ were incubated at room temperature with 3 replicates, and the samples were taken after 24 h, 3 days, and 6 days. Nutrient solutions were continuously aerated during the incubation period.

The samples were taken from well-shaken nutrient solutions using 10 mL syringes and filtered through 0.02 μm filters (inorganic membrane filter Anotop 25 Plus, Whatman-GE Healthcare Life Sciences, Germany). The filters were attached to the syringes and filtrates were obtained by applying gentle pressure on the syringes. The filter size was selected after practically determining that Fe precipitates were able to pass through pore size bigger than 0.02 μm, i.e., through filter MN 619 G1/4, Macherey-Nagel. The clear filtrates (11 g (10 mL approximately) filtrate +1.2 g 1:1 diluted HNO_3_) from all the treatments were diluted 10-folds with 3.2 M HNO_3_ and were analyzed for nutrient concentration using ICP-OES (Agilent 5110, Synchronous Vertical Dual View (SVDV), Agilent, USA). Details of ICP-OES analysis: method, traces (MES 1, 2, 3); sample solution matrix, 3.2 M HNO_3_; internal standard, scandium (1 mg L^–1^) online mix; tubing, PVC; nebulizer, MiraMist PFA; torch, 1.8 mm quartz. The precipitated minerals from the nutrient solutions were isolated on the sixth day of incubation. For better understanding of the factors influencing the stability of nutrient solutions, the identity of the crystalline material formed and the composition of the precipitates were determined using XRD (Bruker AXS D8 reflection diffractometer with Cu Kα radiation, Germany) and SEM-EDX (Zeiss Sigma 300 FEG-SEM, Germany) respectively.

#### 2.4.1 XRD Analysis

The mineral precipitates were isolated by filtration through 0.45 μm regenerated cellulose membrane filters (50 mm diameter; Whatman, GE Healthcare Life Sciences). The samples were dried under a vacuum at 40 °C, ground with a mortar and pestle, and mounted on a standard powder XRD sample holder. The diffractogram was recorded using a BrukerAXS D8 reflection diffractometer with Cu Kα radiation. Generator settings were 40 kV and 40 mA, soller slits 2.5° and 15 mm fixed sample irradiation, measuring range: 2θ = 5 – 70.0° with Lynxeye_XE_T (1d mode) detector, measuring time 0.25 s/step. An antiscatter knife was used (automatic). The databases searched for the model compounds are the inorganic crystal structure database and the crystallography open database.

#### 2.4.2 SEM-EDX Measurement

The ground precipitates (section 2.4.1) were mounted on carbon tape and put on a standard SEM-stub. SEM was done on a Zeiss Sigma 300 FEG-SEM adjusted to Secondary Electron (SE) 20 kV (WD 10 mm). The samples were not carbon-coated to prevent charging. SEM-EDX mappings were collected using the Oxford Instruments X-Max 80 mm^2^ EDX detector at 20 kV. Elemental quantification using SEM-EDX was done without the use of a standard using Oxford Aztec software and should be considered as an indication of the composition (in weight%). EDX is a semiquantitative technique and probes only the outer few micrometers of a sample.

### 2.5 Statistical Analysis

The data were analyzed by two-way ANOVA using Statistix 10 software. The significance of differences among the individual treatments was determined using the least significant difference (LSD) test at the probability level of 0.05.

## 3 Results

### 3.1 Experiment with Fava Bean

#### 3.1.1 Growth and Chlorophyll Contents of Fava Bean

The growth of fava bean plants was adversely affected by an increase in alkalinity levels (Figure 1). Compared to control, shoot dry matter remained unaffected at 5 mM NaHCO_3_, but it reduced to about one-half at 15 mM NaHCO_3_ (Figure 1A,B and Table 1). There was no difference in shoot dry matter between nonchelated and chelated micronutrients (Figure 1C–E, Table 1). Similarly, root dry matter remained unaffected at 5 mM NaHCO_3_ but declined significantly at 15 mM NaHCO_3_ under both micronutrient sources (Table 1). Moreover, the root dry matter did not follow any specific response related to the micronutrient sources. The chlorophyll contents of fava bean plants were not affected by alkalinity, but plants supplemented with chelated micronutrients had lower chlorophyll contents than those supplied with nonchelated micronutrients (Table 1).

**Figure 1:**
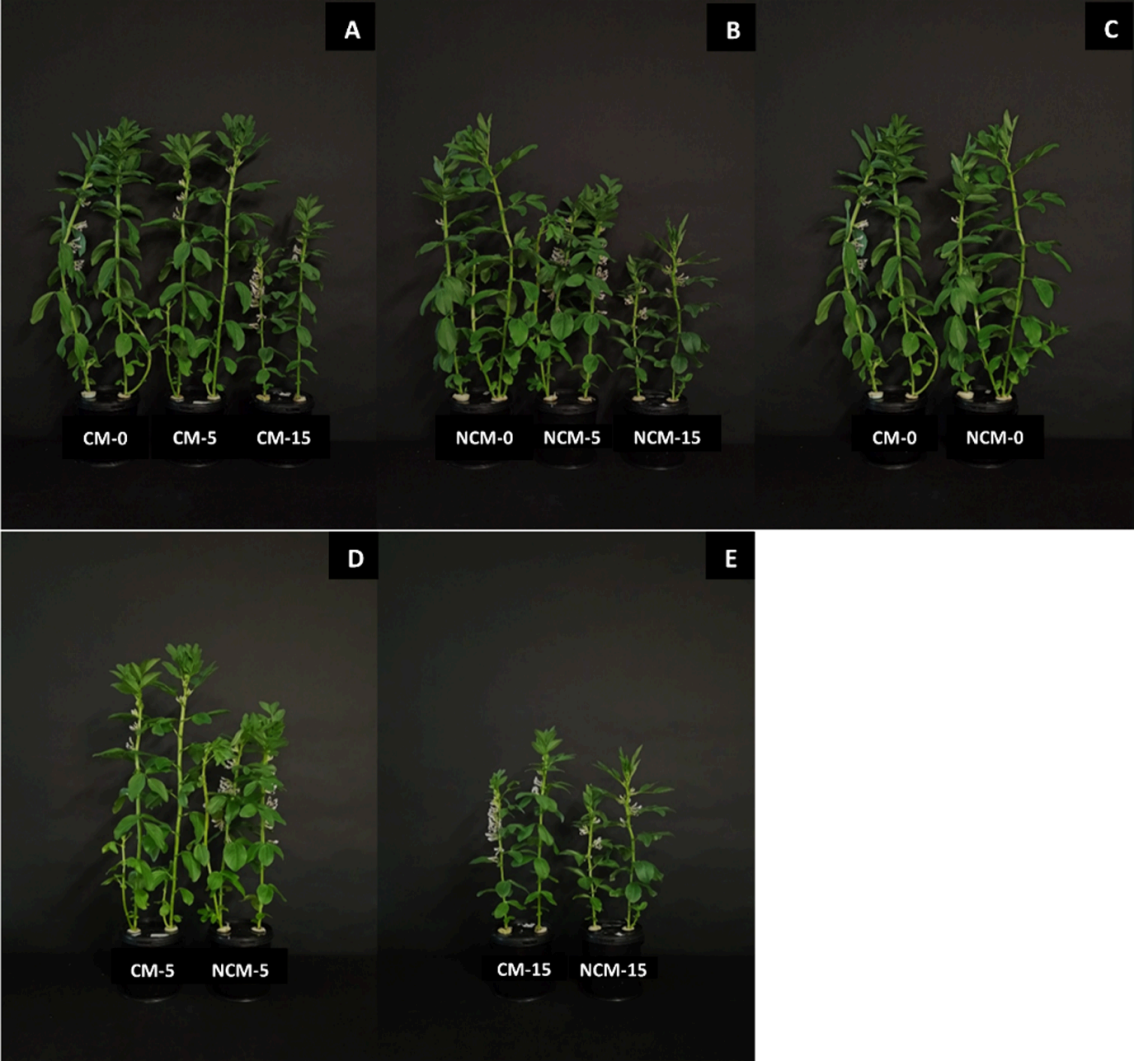
Visual appearance of 4-week-old fava bean plants grown in hydroponics: CM and NCM represent EDTA-chelated micronutrients (Cu, Zn, and Mn) and nonchelated micronutrients, respectively, while 0, 5, and 15 represent NaHCO_3_ levels (mM). (A) Different alkalinity levels with CM. (B) Different alkalinity levels with NCM. (C) CM vs NCM at 0 mM NaHCO_3_. (D) CM vs NCM at 5 mM NaHCO_3_. (E) CM vs NCM at 15 mM NaHCO_3_.

**Table 1:** Shoot and Root Dry Biomasses and Chlorophyll Contents of Four-Week-Old Fava Bean Plants Grown with Either Nonchelated or Chelated Cu, Zn, and Mn under Different Alkalinity Levels[Table-fn t1fn2]

alkalinity (NaHCO_3_)	micronutrients (Cu, Zn, Mn)	shoot dry biomass (g pot^–1^)	root dry biomass (g pot^–1^)	chlorophyll contents, SPAD value
0 mM	nonchelated	10.4a	4.6a	41.6b
	chelated	10.2a	4.0b	39.0c

5 mM	nonchelated	9.9a	4.3ab	45.0a
	chelated	10.3a	4.1b	41.0bc

15 mM	nonchelated	5.8b	3.9b	45.3a
	chelated	5.7b	3.2c	42.4b

CV		11.07	7.71	3.20
LSD_0.05_		1.45	0.47	2.05

a Values are means of four independent pot replicates. The values sharing different letters for each parameter are significantly (*p* ≤ 0.50) different from each other.

#### 3.1.2 Nutrient Concentrations in Plant Tissues of Fava Bean

The concentrations of Fe, P, and Zn in shoots supplied with chelated micronutrients were at least 21%, 16% and 14% higher, respectively, than those supplied with nonchelated micronutrients (Figure 2A,C,G). The shoot Mn concentrations of the chelated micronutrient supplied plants were 20% and 26% higher at 0 and 5 mM NaHCO_3_, respectively than nonchelated micronutrient supplied plants, but such positive effect of chelated micronutrients on shoot Mn concentration was not recorded at 15 mM NaHCO_3_ (Figure 2E).

**Figure 2:**
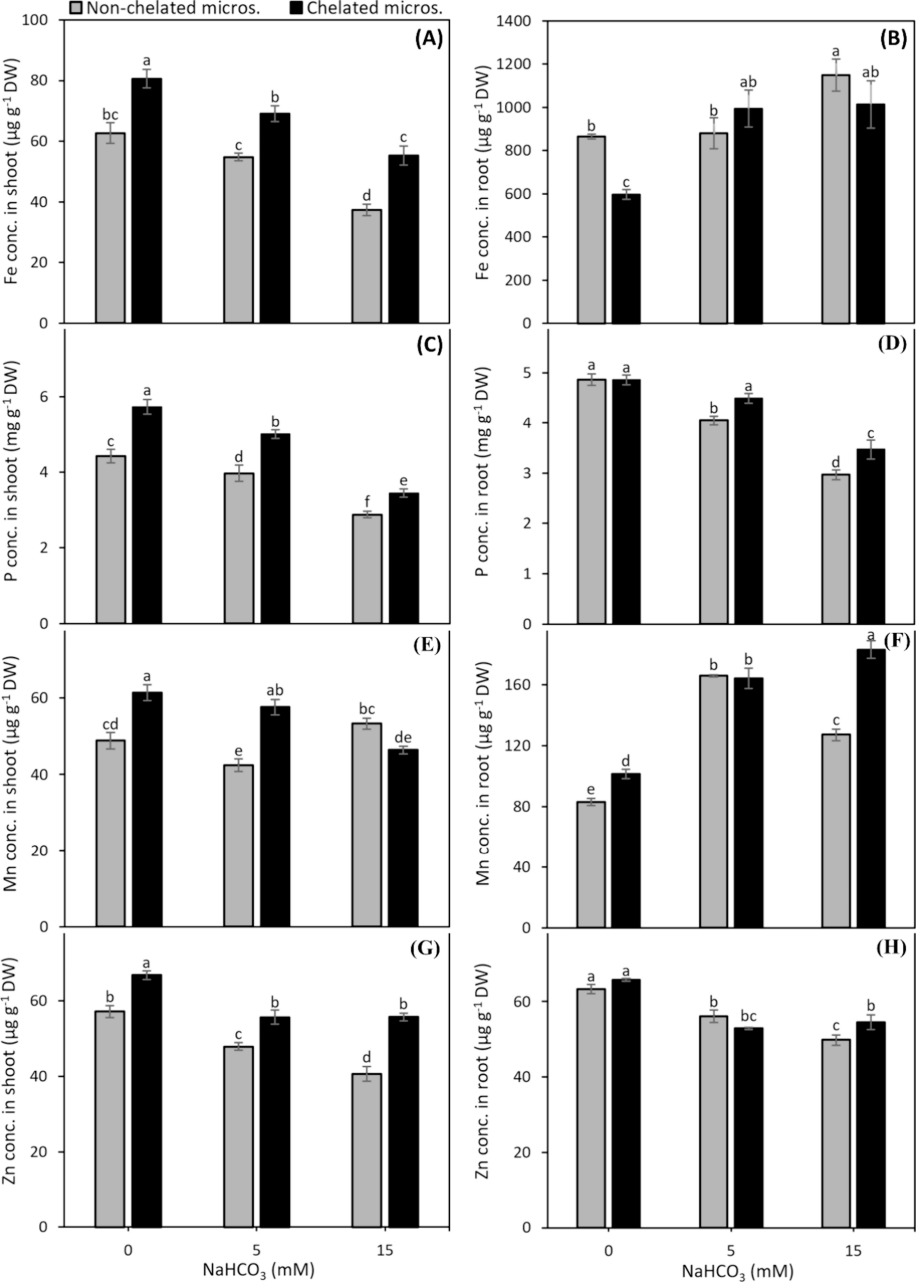
Concentration of Fe (A,B), P (C,D), Mn (E,F), and Zn (G,H) in shoot and root, respectively, of fava bean plants grown in nutrient solution with either nonchelated or chelated Cu, Zn, and Mn under various alkalinity levels. Bars indicate means ± SE of 4 replicates.

The root Fe concentration of chelated micronutrients supplied plants was either slightly lower than or did not differ from the plants supplied with nonchelated micronutrients (Figure 2B). In comparison to nonchelated micronutrients, the root P concentration was significantly higher with chelated micronutrients at 5 and 15 mM NaHCO_3_ (Figure 2D). The use of chelated micronutrients generally resulted in a higher Mn concentration in roots than nonchelated micronutrients (Figure 2F). The root Zn concentration with chelated micronutrients was higher at 15 mM NaHCO_3_, whereas, it remained similar to nonchelated micronutrients at 0 and 5 mM NaHCO_3_ (Figure 2H).

The shoot Cl^–^ and PO_4_^3–^ concentrations were decreased with increase in alkalinity, and were 22% and 31%, respectively lower under nonchelated micronutrients compared to chelated micronutrients (Figure 3A,G). The alkalinity-induced decline in NO_3_^–^ concentration under chelated micronutrients was observed only at 15 mM NaHCO_3_, where NO_3_^–^ was yet at least 163% higher than that nonchelated micronutrients (Figure 3C). The shoot SO_4_^2–^ concentration was at least 38% higher at 0 mM as compared to 5 and 15 mM NaHCO_3_, whereas it remained uninfluenced by the micronutrient sources (Figure 3E).

**Figure 3:**
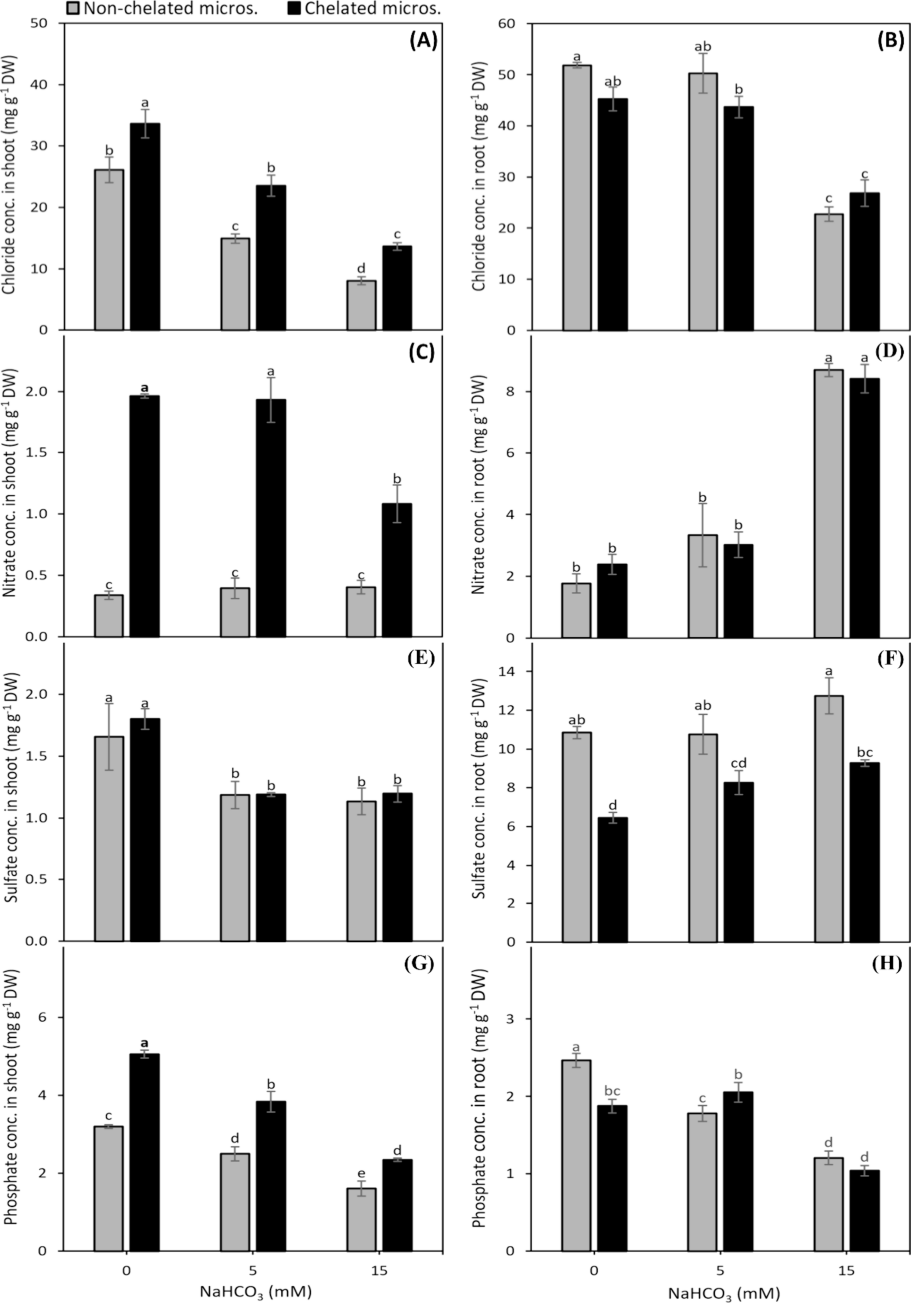
Effect of nonchelated and chelated Cu, Zn, and Mn on chloride (A,B), nitrate (C,D), sulfate (E,F), and phosphate (G,H) concentration in shoot and root, respectively, of fava bean plants under different alkalinity levels. Bars indicate means ± SE of 4 replicates.

The root Cl^–^ concentration was declined by about one-half whereas of root NO_3_^–^ concentration increased by about 3 times at 15 mM NaHCO_3_ as compared to 0 and 5 mM NaHCO_3_. However, the root Cl^–^ and NO_3_^–^ concentrations remained unaffected by the micronutrient sources at all alkalinity levels (Figure 3B,D). The root SO_4_^2–^ concentration was not much affected by alkalinity level, whereas it at least 23% lower with chelated micronutrients compared to nonchelated micronutrients (Figure 3F). The root PO_4_^3–^ concentration was declined by alkalinity treatments whereas it remained unaffected by the micronutrients’ sources (Figure 3H).

#### 3.1.3 Accumulation of Organic Compounds in Plant Tissues of Fava Bean

In general, the accumulation of malate, oxalate, and citrate in shoot and root increased with increase in alkalinity. The malate concentration in shoot remained uninfluenced by the micronutrient source (Figure 4A) whereas of oxalate and citrate were 1.6 and 1.3 fold, respectively higher with chelated micronutrients than that with nonchelated micronutrients (Figure 4C,E). In contrast, the accumulation of malate, oxalate, and citrate in roots was 2.1-, 5.3-, and 2-fold, respectively, higher with nonchelated micronutrients than with chelated micronutrients (Figure 4B,D,F).

**Figure 4:**
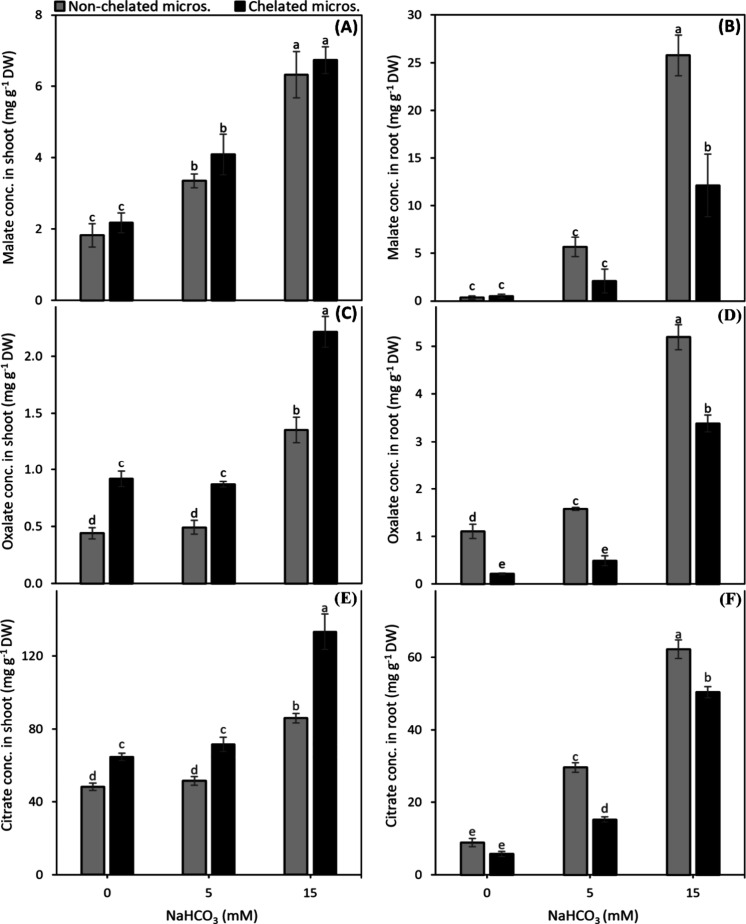
Effect of nonchelated and chelated Cu, Zn, and Mn on malate (A,B), oxalate (C,D), and citrate (E,F) concentration in shoot and root, respectively, of fava bean plants under different alkalinity levels. Bars indicate means ± SE of 4 replicates.

#### 3.1.4 H_2_O_2_ and MDA Concentration

The leaf H_2_O_2_ concentration was increased by alkalinity treatments; however, the increase was 8% and 7% less under chelated micronutrients than nonchelated micronutrients at 5 and 15 mM NaHCO_3,_ respectively (Table 2). Accordingly, the alkalinity-induced increase in leaf MDA concentration was 7% and 6% less than nonchelated micronutrients at 5 and 15 mM NaHCO_3_ (Table 2).

**Table 2:** Effect of Nonchelated and Chelated Cu, Zn, and Mn on Leaf H_2_O_2_ and MDA Concentration of Fava Bean Plants under Different Alkalinity Levels[Table-fn t2fn2]

alkalinity (NaHCO_3_)	micronutrients (Cu, Zn, Mn)	leaf H_2_O_2_ concentration (μg g^–1^ FW)	leaf MDA concentration (μg g^–1^ FW)
0 mM	nonchelated	1.4e	5.7e
	chelated	1.4e	5.5e

5 mM	nonchelated	1.9c	7.5c
	chelated	1.8d	7.0d

15 mM	nonchelated	2.4a	8.5a
	chelated	2.2b	8.1b

CV		4.28	2.04
LSD_0.05_		0.119	0.216

a Values are means of four independent pot replicates. The values sharing different letters for each parameter are significantly (*p* ≤ 0.50) different from each other.

### 3.2 Experiment with Maize Plants

#### 3.2.1 Growth of Maize Plants

Plant growth was significantly reduced by alkalinity (Figure 5A,B); however, this growth reduction was independent of the micronutrient source (Figure 5C–E). Shoot dry weight of maize plants at 0 mM NaHCO_3_ was 7-fold and 23-fold higher than at 5 and 15 mM NaHCO_3_, respectively (Table 3). Similarly, the root dry weight of maize plants at 0 mM NaHCO_3_ was 3-fold and 13-fold higher than those at 5 and 15 mM NaHCO_3_, respectively (Table 3).

**Figure 5:**
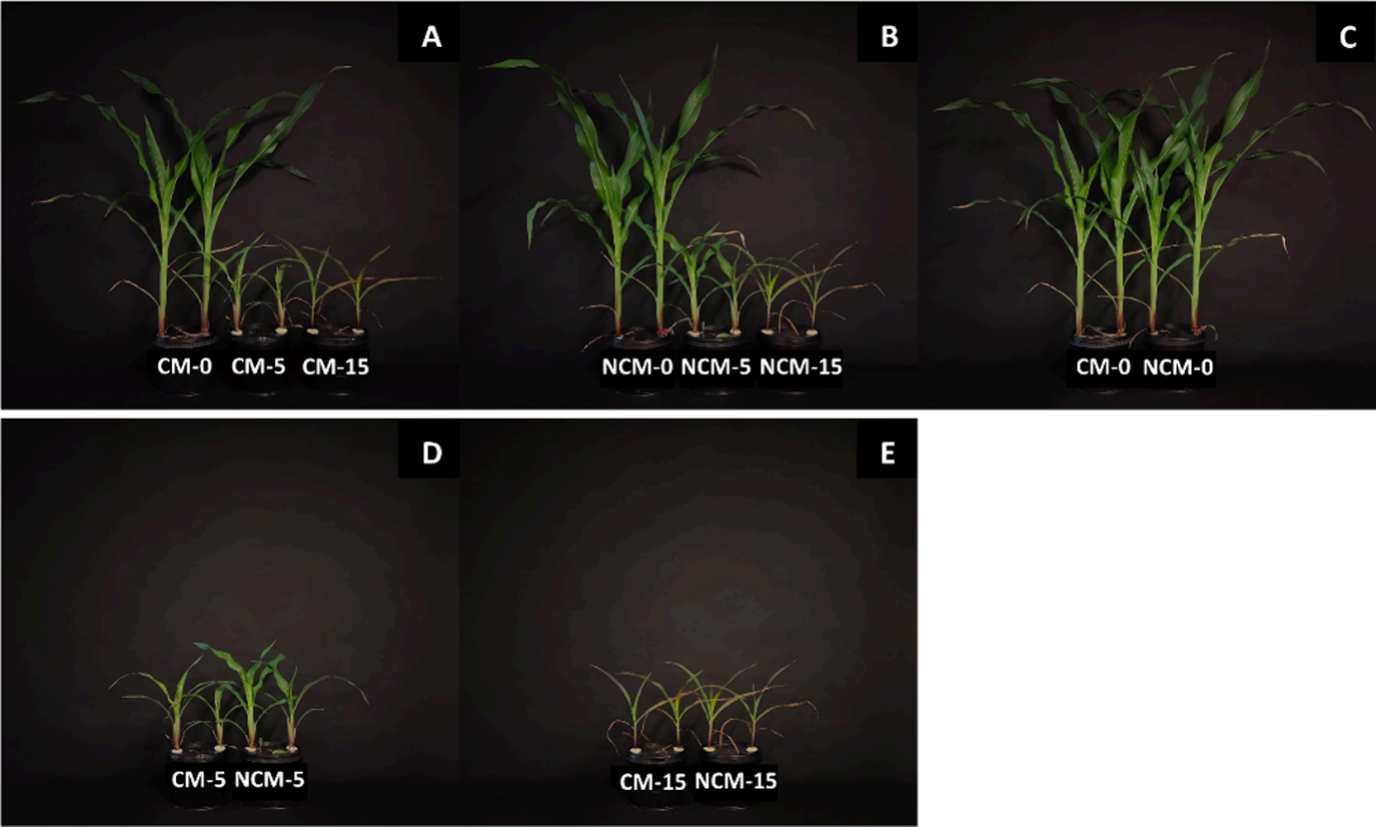
Visual appearance of 4-week-old maize plants grown in hydroponics: CM and NCM represent EDTA-chelated micronutrients (Cu, Zn, and Mn) and nonchelated micronutrients, respectively, while 0, 5, and 15 represent NaHCO_3_ levels (mM). (A) Different alkalinity levels with CM. (B) Different alkalinity levels with NCM. (C) CM vs NCM at 0 mM NaHCO_3_. (D) CM vs NCM at 5 mM NaHCO_3_. (E) CM vs NCM at 15 mM NaHCO_3_.

**Table 3:** Shoot and Root Dry Biomasses of Four-Week-Old Maize Plants Grown with Either Nonchelated or Chelated Cu, Zn, and Mn under Different Alkalinity Levels[Table-fn t3fn2]

alkalinity (NaHCO_3_)	micronutrients (Cu, Zn, Mn)	shoot dry biomass (g pot^–1^)	root dry biomass (g pot^–1^)
0 mM	nonchelated	32.2a	9.8a
	chelated	32.8a	9.7a

5 mM	nonchelated	5.9b	3.4a
	chelated	4.0bc	3.1b

15 mM	nonchelated	1.4c	0.8c
	chelated	1.4c	0.7c

CV		13.99	9.33
LSD_0.05_		3.30	0.78

a Values are means of four independent pot replicates. The values sharing different letters for each parameter are significantly (*p* ≤ 0.50) different from each other.

#### 3.2.2 Nutrients’ Concentration in Shoot and Root of Maize Plants

The shoot Fe and Mn concentrations were increased by increase in alkalinity level (Figure 6A,E). A similar trend for shoot P concentration was observed, except for a lower P concentration at 15 mM NaHCO_3_ as compared to the nonalkaline control. The chelated micronutrients improved the shoot Fe, P and Mn concentrations at 5 mM NaHCO_3_ by 10, 15 and 81%, respectively compared to nonchelated micronutrients (Figure 6A,C,E). The shoot Zn concentration was neither affected by alkalinity nor by micronutrient source (Figure 6G).

**Figure 6:**
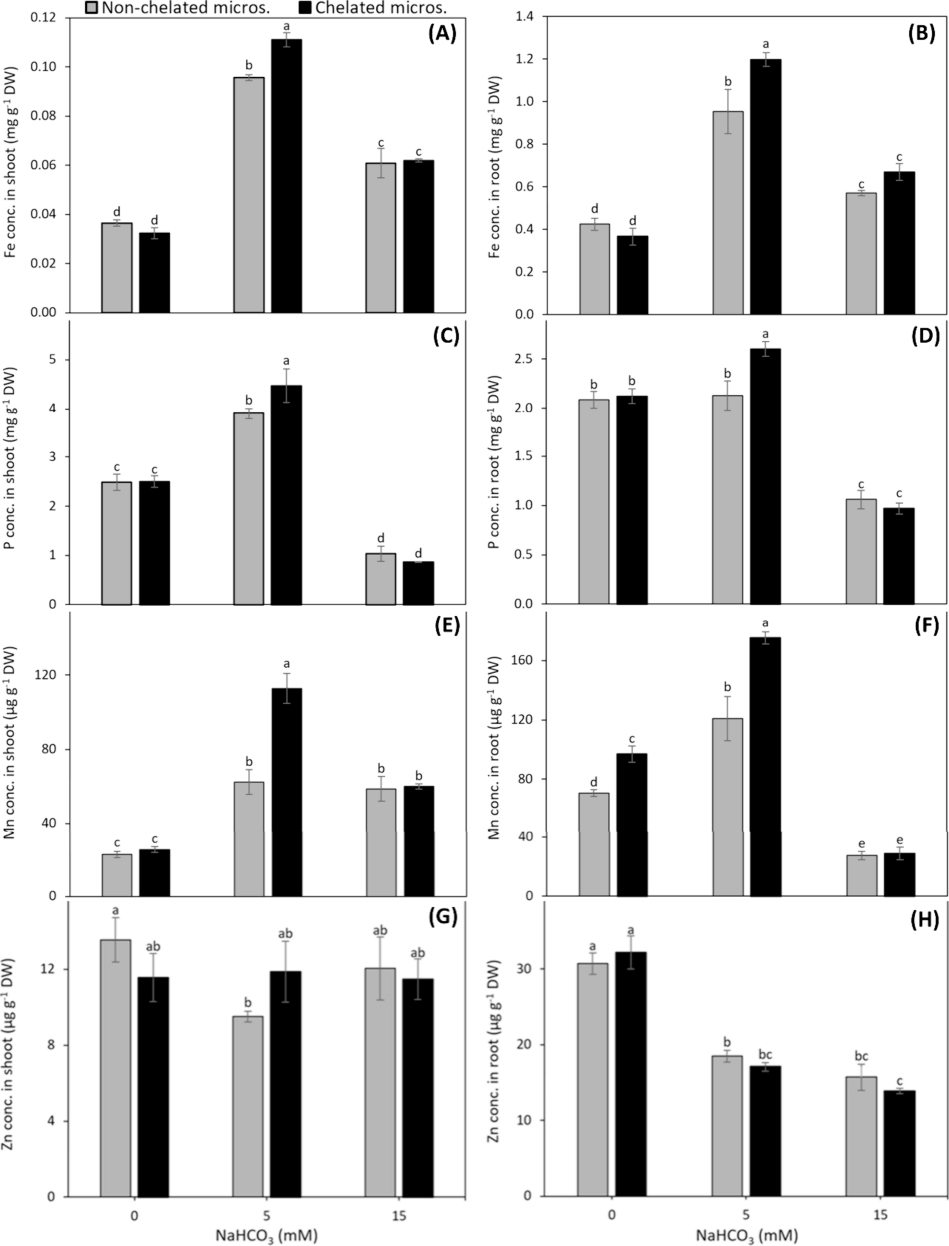
Concentration of Fe (A,B), P (C,D), Mn (E,F), and Zn (G,H) in shoot and root, respectively, of 4-week old maize plants grown with either nonchelated or chelated Cu, Zn, and Mn under different alkalinity levels. Bars indicate means ± SE of 3 replicates.

At 5 mM NaHCO_3_, the root Fe and Mn concentrations were higher than those under the control, and were 26% and 45% higher, respectively with chelated micronutrients than that with nonchelated micronutrients (Figure 6B,F). The root P concentration at 15 mM NaHCO_3_ and the root Zn concentration at 5 and 15 mM NaHCO_3_ were decreased to one-half as compared to the nonalkaline control, but these were not much affected by the source of micronutrients (Figure 6D and 6H).

### 3.3 Speciation Simulation Study of Nutrients

The speciation differences among the three levels of NaHCO_3_ additions were very small, because of the small differences in the ionic strength among the solutions. Therefore, only the results for 15 mM NaHCO_3_ level are presented, but the data for all three levels of NaHCO_3_ addition are given in the Supporting Information.

The chelated proportions of the metals (Cu, Fe, Mn, Zn, Ca, Mg) relative to their total concentrations for the nonfully and the fully chelated micronutrient solutions are presented in parts A and B of Figure 7, respectively. The vertical red lines represent the original experimental pH values at 0, 5, and 15 mM NaHCO_3_, respectively. As expected, in the nonfully chelated micronutrient solutions, Fe was replaced by Cu, Zn, and Mn ions with increasing pH but only a very small part of Fe (<1%) was lost by precipitation. In nonfully chelated system, Cu remained fully chelated above approximately pH 5, Zn above approximately pH 6.5 and Mn above approximately pH 7.5 whereas Ca and Mg ions did not get chelated in this system (Figure 7A). In the fully chelated micronutrient-containing solution, Cu and Zn remained in the completely chelated forms within the pH range of 4–10 while Fe remained completely chelated up to approximately pH 6.5 (Figure 7B).

**Figure 7:**
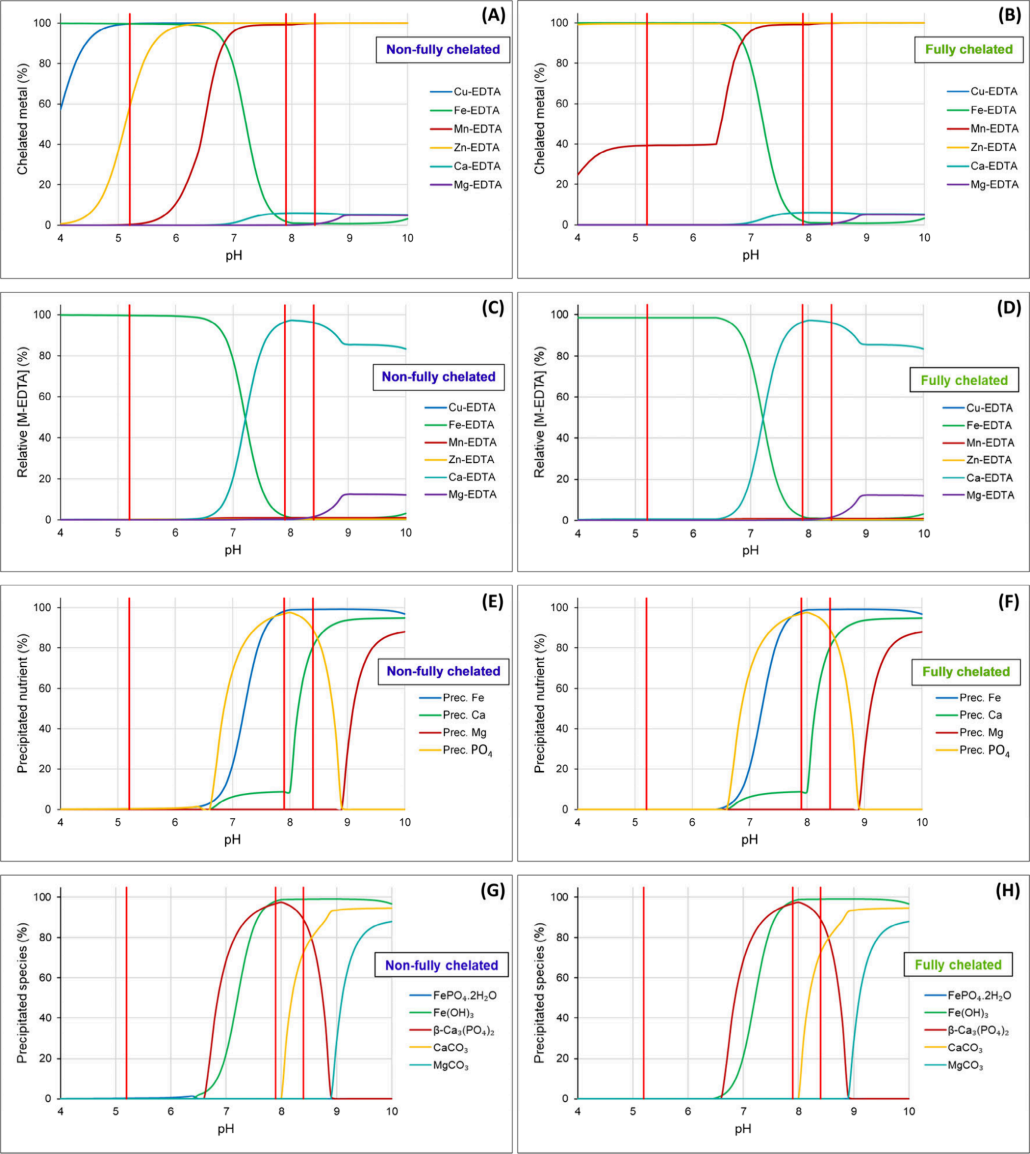
Proportion (%) of different micronutrients bound to EDTA (A,B), the concentration of each metal–EDTA complex relative to the total concentration of EDTA (C,D), precipitated nutrients (E,F) and their precipitated species (G,H) in nutrient solution with nonchelated and chelated Cu, Zn, and Mn, respectively, at high pH.

The concentration of each metal–EDTA complex relative to the total concentration of EDTA for the nonfully and fully chelated micronutrient solutions is presented in Figure 7C,D, respectively. In the nonfully chelated micronutrient solutions, the major replacement of Fe from its complex only started when competition by Ca became significant, above approximately pH 6.5. However, the micronutrients replaced Fe at lower pH values and were completely chelated without significant loss of the Fe-EDTA complex (Figure 7C). At pH values <6.5, approximately 98.6% of EDTA was chelated with Fe, and remaining 1.4% of EDTA that was introduced with Cu, Mn, and Zn was chelated to these micronutrients and Ca (Figure 7D).

The overall precipitated fraction of nutrients relative to the concentration of the nutrient ions is shown in Figure 7E,F. No precipitates were found involving K, NH_4_^+^, Cl, NO_3_^–^, SO_4_^2–^, B, Cu, Mn, Mo, or Na ions (Figure 7G,H). At pH 5.2, only a negligible and insignificant amount of Fe and PO_4_^3–^ (0.3% for each) was lost by precipitation as strengite (FePO_4_·2H_2_O) in the nonfully chelated nutrient solution (Figure 7G). However, in the fully chelated nutrient solution, no precipitates were recorded below approximately pH 6.5. At pH 7.9, most of Fe (98%) was precipitated as iron hydroxide (Fe­(OH)_3_), and most of PO_4_^3–^ (97%) and a significant part of Ca (9%) were precipitated as tricalcium phosphate (TCP, β-Ca_3_(PO_4_)_2_). Above pH 8.0, the precipitation of calcite is expected to start. At pH 8.4, almost all Fe (99%) was precipitated as iron hydroxide (Fe­(OH)_3_), most of PO_4_^3–^ (89%) as TCP (β-Ca_3_(PO_4_)_2_) and most of Ca (80%) as calcite (CaCO_3_) and TCP (β-Ca_3_(PO_4_)_2_). As the precipitation of CaCO_3_ effectively removes soluble Ca from the solution, the solubility of TCP (β-Ca_3_(PO_4_)_2_) will increase, reducing the relative amount of precipitated PO_4_^3–^ (Figure 7H).

No significant precipitation is expected in the nutrient solution at pH 5.2; only 0.3% of Fe and PO_4_^3–^ could be lost from the nonfully chelated nutrient solution, but no precipitation is expected in the fully chelated nutrient solution. At pH 7.9, most Fe and PO_4_^3–^ and a significant part of Ca are expected to precipitate in both nutrient solutions and even more at pH 8.4.

### 3.4 Incubation Experiment

#### 3.4.1 Concentration of Soluble Fe and P in Nutrient Solutions

The nutrient solution containing Fe-EDTA and Fe-DTPA got turbid (Figure 8A,B) in the presence of 5 mM NaHCO_3_ alkalinity and turbidity got denser with the passage of time. However, this effect was not observed in Fe-HBED containing a nutrient solution (Figure 8C).

**Figure 8:**
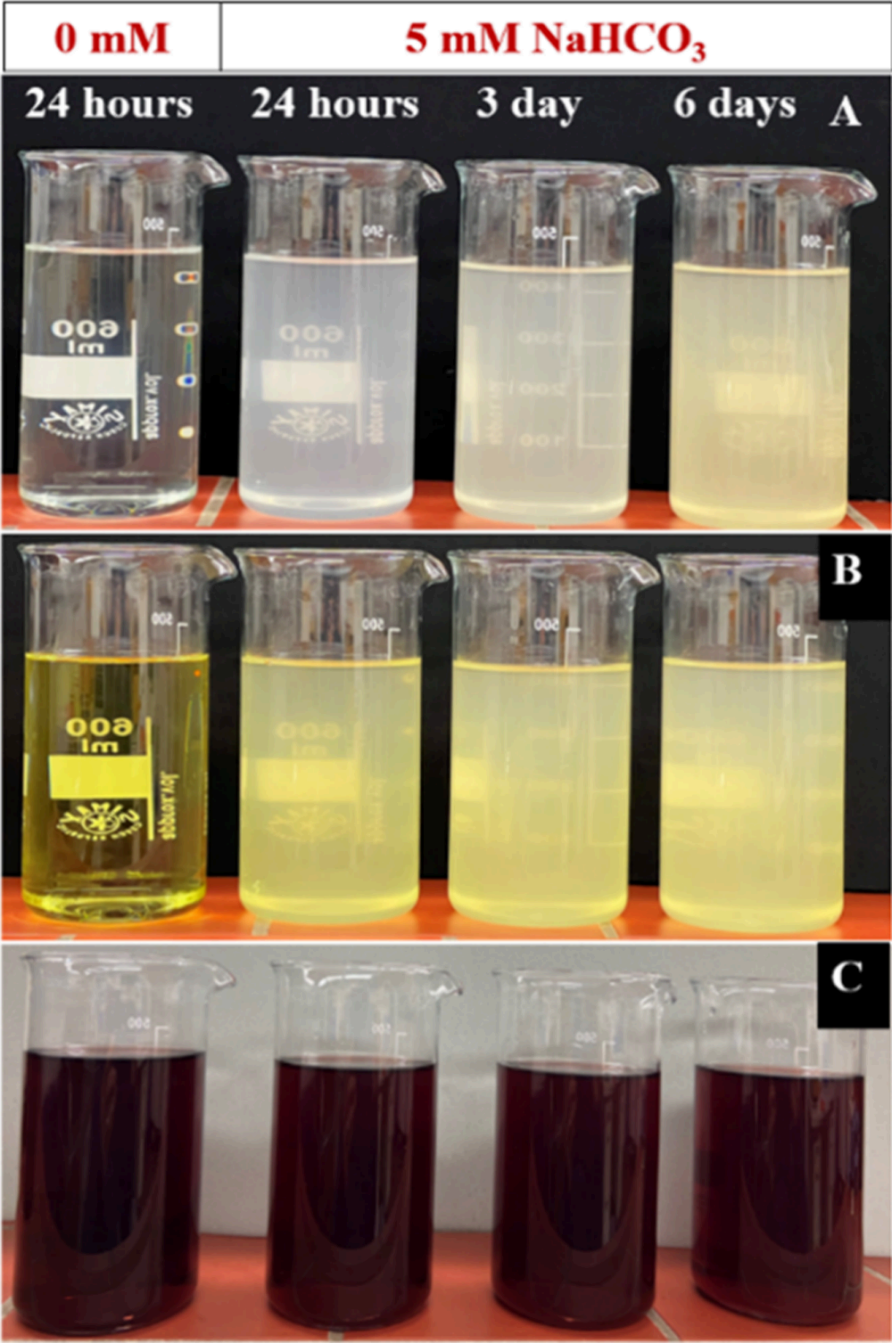
Visual condition of nutrient solutions containing Fe-EDTA (A), Fe-DTPA (B), and Fe-HBED (C) at 0 mM NaHCO_3_ after 24 h and at 5 mM NaHCO_3_ after 1, 3, and 6 days of incubation.

In nonalkaline nutrient solution (control), the concentration of soluble Fe did not decline with the passage of time across all the chelating agents, though it was substantially higher in Fe-HBED containing nutrient solution than those containing Fe-EDTA and Fe-DTPA (Table 4). Moreover, the concentration of soluble Fe in Fe-HBED containing nutrient solution was neither declined with increase in the level of NaHCO_3_ alkalinity nor with increase in the incubation duration (Table 4). However, the concentration of soluble Fe in Fe-EDTA and Fe-DTPA containing nutrient solutions progressively declined with the increase in NaHCO_3_ alkalinity as well as the incubation duration, depending upon the alkalinity level. The concentration of soluble Fe in Fe-EDTA containing nutrient solution was declined by 11% and 33% after 24 h, 32% and 50% after 3 days, and 52% and 64% after 6 days of incubation under 5 mM and 15 mM NaHCO_3_ alkalinity, respectively, compared to nonalkaline control. Similarly, the concentration of soluble Fe in Fe-DTPA containing nutrient solution was declined by 25% and 46% after 24 h, 33% and 45% after 3 days, and 42% and 48% after 6 days of incubation under 5 mM and 15 mM NaHCO_3_ alkalinity, respectively.

**Table 4:** Percentage (%) of Soluble Fe and P in Alkaline Nutrient Solutions Containing Different Fe Chelates[Table-fn t4fn2]

		Fe			P		
Fe source	alkalinity (NaHCO_3_)	24 h	3 days	6 days	24 h	3 days	6 days
Fe-EDTA	0 mM	89.7de	89.5de	90.6cd	82.5a	90.7a	89.0a
Fe-DTPA		87.1f	88.1ef	92.4c	83.3a	88.5a	89.8a
Fe-HBED		97.0ab	96.1ab	96.3ab	83.3a	85.1a	87.2a

Fe-EDTA	5 mM	78.7g	57.5i	38.7m	43.6c	0.0e	0.0e
Fe-DTPA		62.4h	55.2i	50.4j	35.7d	0.0e	0.0e
Fe-HBED		96.7ab	95.4b	96.1ab	58.1b	45.4c	39.9c

Fe-EDTA	15 mM	56.4i	39.0m	26.4n	0.0e	0.0e	0.0e
Fe-DTPA		41.5l	43.1kl	44.7k	0.0e	0.0e	0.0e
Fe-HBED		96.1ab	96.3ab	97.9a	0.0e	0.0e	0.0e

CV			1.96			13.86	
LSD_0.05_			0.265			0.516	

a Values are means of three independent replicates. The values sharing different letters for Fe or P are significantly (*p* ≤ 0.50) different from each other.

In nonalkaline nutrient solution, the concentration of soluble P did not change with the passage of time for all Fe chelates (Table 4). At 5 mM NaHCO_3_ alkalinity, the concentration of soluble P in Fe-EDTA and Fe-DTPA containing nutrient solution declined by more than half after 24 h, and dropped to zero after 3 days of incubation (Table 4). However, in Fe-HBED containing nutrient solution at 5 mM NaHCO_3_ alkalinity, the soluble P was still half of what was recorded in nonalkaline control even after 6 days of incubation. Contrarily, at 15 mM NaHCO_3_ alkalinity, there was absolutely no soluble P in any of the Fe-chelated added nutrient solutions.

#### 3.4.2 Concentration of Soluble Ca and Mg in Nutrient Solutions

There was nonsignificant decline in soluble concentration of Ca and Mg at 5 mM NaHCO_3_ as compared to nonalkaline control. However, at 15 mM NaHCO_3_, soluble Ca and Mg concentrations were declined by 11% and 10%, respectively after 24 h of incubation. Compared to nonalkaline control, the decline in soluble Ca concentration of Fe-HBED containing nutrient solution at 15 mM NaHCO_3_ was at-least 1%, 29% and 10% lesser than Fe-EDTA and Fe-DTPA containing nutrient solution after 24 h, 3 and 6 days of incubation, respectively (Table 5).

**Table 5:** Percentage (%) of Soluble Ca and Mg in Alkaline Nutrient Solutions Containing Different Fe Chelates^a^

		Ca			Mg		
Fe source	alkalinity (NaHCO_3_)	24 h	3 days	6 days	24 h	3 days	6 days
Fe-EDTA	0 mM	91.3a	91.3a	89.1ab	95.5a	94.9ab	94.4abc
Fe-DTPA		89.7ab	87.2bcd	87.8a–d	93.9bcd	93.5cde	92.6efg
Fe-HBED		89.3ab	87.8a–d	88.4abc	92.8def	92.4ef	92.1efg

Fe-EDTA	5 mM	85.0c–f	82.8e–h	79.9gh	92.6def	92.6fg	90.2i
Fe-DTPA		83.9d–g	81.1fgh	81.8fgh	91.8fgh	91.8ghi	90.3i
Fe-HBED		86.0b–e	82.3e–h	82.2e–h	92.3ef	92.3ghi	90.5hi

Fe-EDTA	15 mM	79.8h	24.3jk	18.4lm	90.2i	85.9k	83.6m
Fe-DTPA		78.8h	22.4kl	17.6m	89.8i	85.1kl	84.4lm
Fe-HBED		79.8h	52.4i	28.1j	89.4ij	88.3j	86.1k

CV			3.39			0.94	
LSD_0.05_			5.30			0.17	

a Values are means of three independent replicates. The values sharing different letters for Ca or Mg are significantly (*p* ≤ 0.50) different from each other.

#### 3.4.3 Nature and Composition of Precipitates Measured by XRD and SEM-EDX

Significant peaks were detected in samples from Fe-EDTA containing nutrient solutions by XRD analysis. At 5 mM NaHCO_3_, completely amorphous material or nanocrystals were formed, whereas, at 15 mM NaHCO_3_, only CaCO_3_ was a crystalline compound (Figure 9).

**Figure 9:**
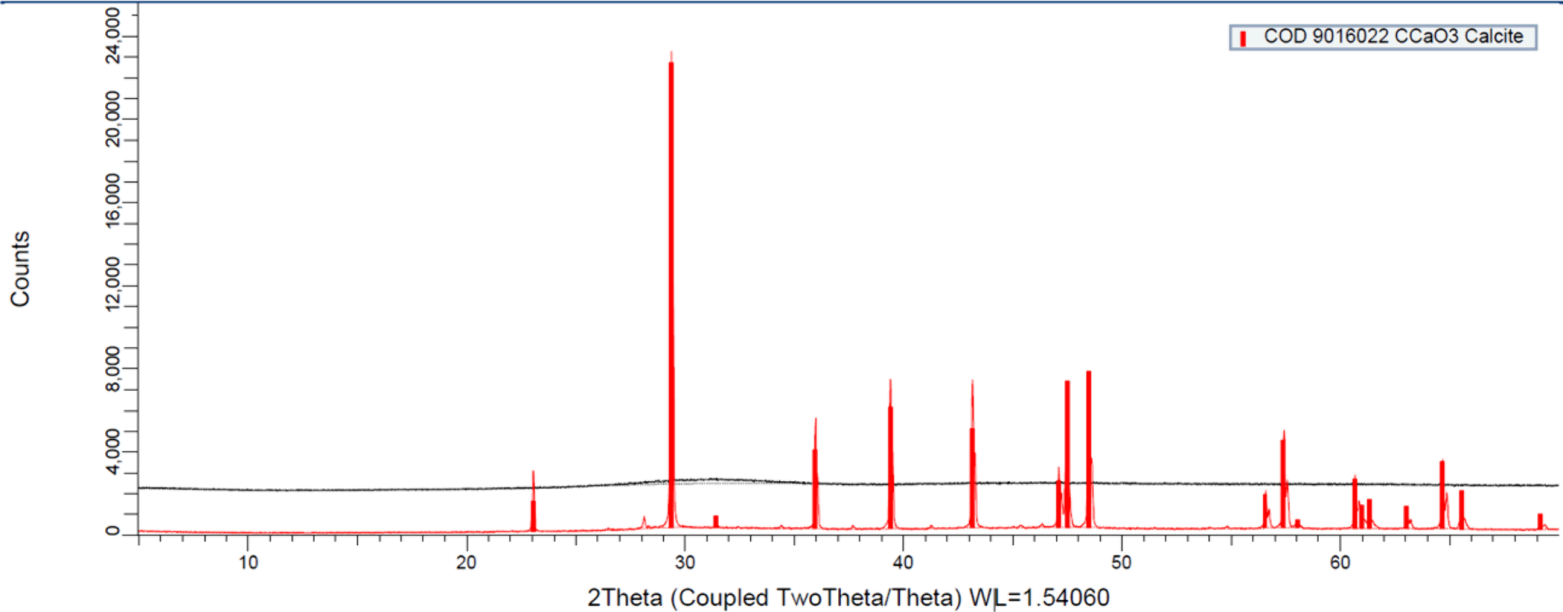
Diffractogram of precipitates collected from Fe-EDTA containing nutrient solution. The black and red lines represent 5 and 15 mM NaHCO_3_ alkalinity levels, respectively.

The precipitates were not homogeneous and consisted of multiple compounds. The different colors in SEM-EDX images represent the regions with differences in composition (Figure 10A,B). At 5 and 15 mM NaHCO_3_ alkalinity, the precipitates from Fe-EDTA and Fe-DTPA containing nutrient solution were mainly comprised of Fe, Ca and P, whereas the main elements in the case of Fe-HBED containing nutrient solution were Ca and P (Table 6 and 7). With respect to the concentrations of Fe and P in precipitates at 5 (Table 6) and 15 mM NaHCO_3_ (Table 7), the chelates followed the order: Fe-HBED < Fe-DTPA < Fe-EDTA.

**Figure 10:**
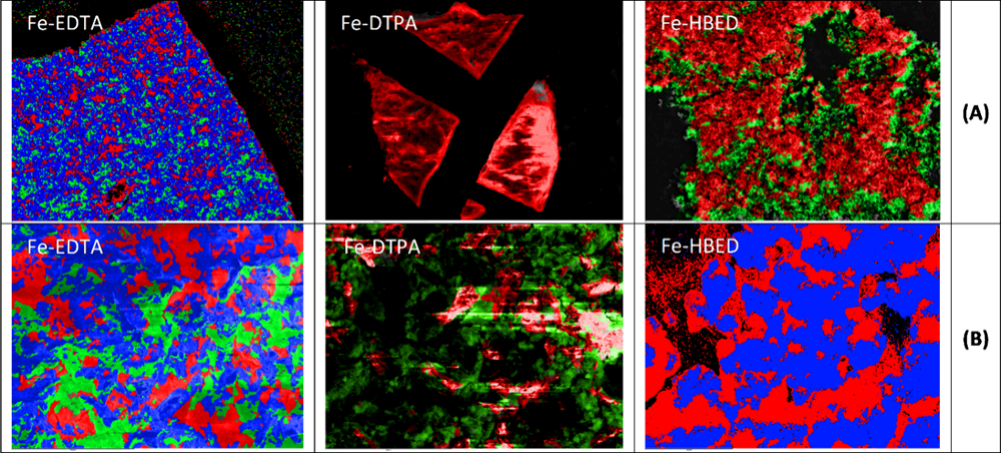
SEM-EDX images of the precipitates collected from the nutrient solution added with different Fe sources containing 5 mM (A) and 15 mM (B) NaHCO_3_. The colors represent the regions with differences in composition.

**Table 6:** Elemental Composition (%) of Precipitates in 5 mM NaHCO_3_ Samples Calculated from SEM-EDX

Fe source	spectrum	C	O	Na	Mg	P	S	K	Ca	Fe	Cu
Fe-EDTA	red	19.0	40.2	-	0.3	10.4	-	-	17.8	12.4	-
	blue	19.2	43.9	-	0.3	10.2	-	0.1	13.8	12.6	-
	green	28.2	41.9	-	0.2	8.4	-	0.1	9.6	11.5	-

Fe-DTPA	red	34.7	38.7	-	0.2	8.1	0.1	0.1	11.2	6.9	0.1

Fe-HBED	green	46.4	39.6	0.3	0.2	4.8	0.3	-	8.1	0.2	0.1
	red	28.9	43.0	0.2	0.4	9.5	0.3	-	17.2	0.5	0.2

**Table 7:** Elemental Composition (%) of Precipitates in 15 mM NaHCO_3_ Samples Calculated from SEM-EDX

Fe source	spectrum	C	O	Na	Mg	P	S	K	Ca	Fe	Cu
Fe-EDTA	red	14.5	32.5	-	0.2	4.9	-	-	32.3	15.6	-
	green	16.7	56.5	-	0.4	4.8	-	-	15.6	6.0	-
	blue	13.5	51.1	-	0.3	2.0	0.1	-	30.1	3.0	-

Fe-DTPA	red	10.7	53.8	-	0.3	2.6	-	-	30.0	2.7	-
	green	9.9	45.4	-	0.4	8.6	-	-	22.5	13.0	0.2

Fe-HBED	red	20.2	31.0	0.1	0.1	0.5	0.1	-	47.6	-	0.4
	blue	16.9	53.2	-	0.2	1.8	-	-	27.7	0.1	-

## 4 Discussion

This study was conducted to investigate the differential effect of chelated and nonchelated micronutrients on Fe uptake by fava bean and maize plants grown in nutrient solution under varying levels of NaHCO_3_ alkalinity. Moreover, this study simulated Fe precipitation and evaluated the soluble Fe concentration from different Fe chelates under varying levels of NaHCO_3_ alkalinity.

### 4.1 Plant Growth-Related Responses

The alkalinity had a negative effect on the growth and dry matter production of fava bean (Figure 1, Table 1) and maize (Figure 5, Table 3). The alkalinity-induced reduction in plant growth is well-known in the literature.^7,33^ However, it is worth noting that fava bean experienced a decline in dry matter production only at 15 mM NaHCO_3_, whereas maize proved highly sensitive to alkalinity, with its dry matter production severely declining even at 5 mM alkalinity, and it barely survived at 15 mM alkalinity. The decline in dry matter production in fava bean was related to over production of H_2_O_2_ and resultant lipid per oxidation (Table 2).^11^ Over production of H_2_O_2_ in fava bean could be explained by Fe concentration in leaves which was well below the critical deficiency limit of 72 μg g^–1^ at 15 mM NaHCO_3_. Second, a higher accumulation of organic acids (Figure 4) could have induced H_2_O_2_ production by the activation of NADPH oxidases and inhibition of PM H^+^-ATPase.^11,34^ Although alkalinity induced increases in H_2_O_2_ production and lipid peroxidation were lower in chelated micronutrient supplied fava bean plants compared to those supplied with nonchelated micronutrients, this effect did not translate to enhanced alkalinity resistance in terms of dry matter production. This discrepancy could be attributed to the fact that, although significant, the differences in H_2_O_2_ production and lipid peroxidation between the chelated and nonchelated micronutrient sources were relatively small. The effect of the chelated micronutrient source could have been significant if the plants had been grown for a longer period of time, which was only 4 weeks in the present study. Compared to chelated micronutrient supply, a relatively higher root dry matter production under nonchelated micronutrient supply is explained by the extensive growth of the roots to access more nutrients under partially nutrient deprived rooting medium.^35^

### 4.2 Ion Homeostasis

The concentrations of P, Fe, Mn, and Zn in shoot of fava bean followed a decreasing trend with alkalinity (Figure 2A,C,E,G), particularly, there was a linear decrease in shoot P and Fe concentrations with increasing alkalinity levels (Figure 2A,C). There appears to be a greater ion imbalance with nonchelated micronutrients at 15 mM NaHCO_3_ alkalinity (Figure 2E), however, only the shoot Fe concentration under nonchelated micronutrients (Figure 2A) was below the critical deficiency limit of 72 μg g^–1^.^36^ The decline in shoot nutrient concentrations under NaHCO_3_ alkalinity was associated with the loss of soluble concentrations of the nutrients due to high-pH induced precipitation. The speciation study confirmed the instability of Fe-EDTA above pH levels of 6.5, where Fe is replaced by Cu, Zn, Mn, and Ca ions (Figure 7A,B), resulting in the immediate precipitation of free soluble Fe as iron phosphate and/or iron hydroxide. This suggests that a more stable source of Fe is indispensable to ensure adequate Fe uptake by plants at higher pH values. Since the cumulative concentration of Cu, Mn and Zn in nutrient solution was very low than Fe, a very small part of Fe (<1%) was lost by precipitation due to their replacement effect. On the other hand, a significant quantity of Ca was present in the nutrient solution, and it mainly contributed to the loss of soluble Fe from the nutrient solution. The most interesting finding of this study is that the use of EDTA-chelated micronutrients improved not only their own concentrations but also those of Fe and P in the shoots of fava bean, compared to the use of nonchelated micronutrients (Figure 2A,C). The question as to why the shoot P concentration was improved by the chelated micronutrients was explained by the speciation and incubation studies.

As pH increases, SO_4_^2–^ and Cl^–^ form soluble complexes with Ca^2+^ and Mg^2+^, PO_4_^3–^ precipitates with Ca^2+^ as Ca_3_(PO_4_)_2_ and NO_3_^–^ remains as a free ion in the solution.^37^ The lower concentrations of SO_4_^2–^, Cl^–^ and PO_4_^3–^, and or unchanged concentration of NO_3_^–^ in the shoots of nonchelated micronutrient-fed plants are attributed to the above mechanisms (Figure 3). A negatively charged environment builds up in the root zone with alkalinity, which favors the precipitation of cations and reduces the uptake of anions by the plants. The higher uptake of anions with chelated micronutrients, as was recorded in this study, is explained by their decreased precipitation with free cations.

In maize, the toxicity of 5 mM NaHCO_3_ alkalinity was too severe to render very poor growth of plants. In this way, the nutrients acquired by roots get concentrated in smaller biomass, and eventually their concentrations increased as compared to nonalkaline control. The severity of the negative effect of 15 mM NaHCO_3_ was even worse, and the physiological behavior of plants seemed totally impaired. Therefore, the nutrient concentrations could have declined due to impaired root uptake process at this alkalinity level. Hence, the nutrient concentrations in maize cannot be directly compared to those in fava bean, which exhibited relative resistance to the studied NaHCO_3_ alkalinity levels. Furthermore, the nutrient concentrations in maize cannot be fully explained by the precipitation study. The chelated micronutrients had clear positive effects on the concentrations of Fe, P, and Mn in maize plants at 5 mM NaHCO_3_ (Figure 6A–F). In contrast, their reduced concentrations observed at 0 mM NaHCO_3_ can be justified by their similar contents in both the shoot and root of the plants. The results give us a clue that the availability of P and Fe is linked with each other or that these are taken up by the roots using the same mechanisms.

### 4.3 Accumulation of Metabolites

The increased shoot accumulation of organic acids under alkalinity stress (Figure 4) is in line with the novel results reported by Sagervanshi et al.^11^ The plants accumulate more organic acids to maintain charge balance under alkalinity, and the organic acid accumulation is thus related to alkalinity resistance in plants.^12,38^ Moreover, plants manufacture more organic acids under deficiencies of P, Fe, Zn, and Mn^39^ and exude them into the rhizosphere to make favorable conditions for the uptake of these nutrients.^40–42,13^ This could partially explain higher organic acid accumulation in roots under nonchelated micronutrients. Thus, the higher organic acid concentration in the roots of nonchelated micronutrient plants could be regarded as the adaptive mechanism of plants to make a pH balance in the rhizosphere for nutrient uptake, which was not much needed in the case of chelated micronutrients. The link between the nutrient deficiency as well as alkalinity, and the concentration of organic acids in the shoot can be seen in Figure 2 and Figure 4. On the other hand, the higher organic acid accumulation in shoots (Figure 4) with chelated micronutrients as compared to nonchelated micronutrients could be due to the efficient conversion of sugars into organic acids, a cell functioning related mechanism driven by the proper nutritional status of plants (Figure 2).

### 4.4 Oxidative Stress Response

Alkalinity treatments increased the leaf H_2_O_2_ and MDA concentrations, but this increment was lower with chelated micronutrients (Table 2). These results can be associated with higher concentrations of Fe, P, and Zn in the shoot by the use of chelated micronutrients (Figure 2A,C,G). The deficiencies of Fe, P, and Zn trigger the production of H_2_O_2_ and MDA, which are common under alkaline conditions.^43–45^ Moreover, alkalinity stress increases the production of H_2_O_2_, which leads to lipid peroxidation of the plasma membrane, resulting in the production of more MDA as a byproduct.^46–48^ Furthermore, it is evident that under stress conditions and toxic elemental concentrations, EDTA reduces MDA and H_2_O_2_ production,^49,50^ probably due to chelation driven improvement in nutrient uptake by plants.

### 4.5 Nutrient Precipitation and Incubation Studies

The speciation calculations assume thermodynamic equilibrium, the achievement of which may require a considerable amount of time (days), as equilibrium for all precipitation reactions is reached gradually. The precipitated quantities of FePO_4_·2H_2_O and Fe­(OH)_3_ are calculated relative to the total concentration of Fe, β-Ca_3_(PO_4_)_2_ relative to the total concentration of PO_4_^3–^, CaCO_3_ relative to the total concentration of Ca, and MgCO_3_ relative to the total concentration of Mg.

The speciation study showed that Fe-EDTA is unstable at high pH, and Fe can be displaced by other nonchelated metal ions from the Fe-EDTA complex (Figure 7A,B). Second, in the presence of nonchelated micronutrients, Fe is more prone to precipitation with PO_4_^3–^ and OH^–^. The incubation study confirmed that the concentration of soluble P in the nutrient solution was linked to the stability of Fe (Table 4). At 5 mM NaHCO_3_, the soluble P fraction, present only in the Fe-HBED-containing nutrient solution, could be attributed to the stability of this chelate.^51–53^ The stability of Fe-HBED chelate might have prevented the precipitation of Fe and P, as precipitation of P with cations like Ca and Fe tends to be highest at this pH level (Figure 7G). This could satisfactorily explain the higher P concentration in fava bean shoots under the chelated micronutrients compared to the nonchelated micronutrients. The SEM-EDX analysis clarified that the precipitation of P is related to the stability of the Fe chelate (Tables 6 and 7).

At high pH, Ca is detrimental to the stability of chelates with intermediate strength.^24^ Besides Fe, Fe-HBED also maintained better soluble Ca concentrations at 15 mM NaHCO_3_ over time (Table 5). Otherwise, Ca is potentially susceptible to precipitation as CaCO_3_ at high pH, which was confirmed by XRD analysis detecting CaCO_3_ as the dominant crystal in the Fe-EDTA-containing nutrient solution at 15 mM NaHCO_3_ (Figure 9). Since Fe-HBED does not appreciably chelate Ca^2+^, the improved soluble Ca concentration can be related to Fe, and not to any Ca-HBED interaction. Iron inhibits the precipitation of Ca salts (like CaCO_3_ or Ca phosphates) by interfering with nucleation or crystal growth processes.^54^

Since the combined concentrations of Cu, Mn, and Zn were only 1.4% of the Fe concentration added as Fe-EDTA, only less than 1% of Fe was lost by precipitation. The quantity of EDTA added with Fe-EDTA far exceeded that added with the other micronutrients. Consequently, Cu remained fully chelated above approximately pH 5, Zn above approximately pH 6.5, and Mn above approximately pH 7.5. In contrast, the EDTA concentration was insufficient to fully chelate the added Ca and Mg, as evident in Figure 7A. Although the other micronutrients start replacing Fe at lower pH values, these do not contribute significantly to the loss of Fe due to their relatively lower concentrations in the nutrient solution (0.15% for Cu, 1.0% for Mn, and 0.25% for Zn). The high concentration of EDTA added as Fe-EDTA in these nutrient solutions can easily chelate all nonchelated micronutrients without significant loss of soluble Fe. As the additional EDTA concentration for the fully chelated nutrient solution is only slightly higher than for the nonfully chelated nutrient solution (2.028 × 10^–4^ M versus 2.000 × 10^–4^ M), the effect on the relative concentrations of metal-EDTA complexes is hardly visible. It can be observed that at pH values <6.5, approximately 98.6% of EDTA was chelated with Fe, because the additional 1.4% of EDTA that was introduced with Cu, Mn, and Zn is chelated to these micronutrients and Ca (Figure 7D).

It is noteworthy that at 5 mM NaHCO_3_ alkalinity, the soluble Fe concentration in the Fe-DTPA containing nutrient solution decreased gradually, albeit to a lesser extent. In contrast, at 15 mM NaHCO_3_ alkalinity, after a sharp decline in soluble Fe concentration, it remained stable after 24 h of incubation. This suggests that Fe in Fe-DTPA containing nutrient solution underwent a rapid chemical reaction with NaHCO_3_, the rate of which was faster at 15 mM NaHCO_3_ and thereby achieved a steady state within 24 h. On the other hand, Fe-EDTA initially maintained higher soluble Fe concentration than Fe-DTPA, which continued to gradually decline over 6 days of incubation, reaching to substantially lower values than that of Fe-DTPA containing nutrient solution. Among the chelates, Fe-HBED was proved superior as it maintained the initial soluble Fe concentration across the studied alkalinity levels over an incubation period of 6 days. However, the concentration of soluble Fe in Fe-EDTA and Fe-DTPA containing nutrient solutions was progressively declined with the increase in NaHCO_3_ alkalinity as well as the incubation duration depending upon the alkalinity level.

This study provides compelling evidence that Fe availability from Fe-EDTA to fava bean and maize plants could be enhanced by using chelated forms of other micronutrients (Zn, Cu, and Mn) as well. In addition, chelated micronutrients also facilitate the uptake of P and Zn by plants. This was especially evident in fava bean, where EDTA-chelated micronutrients mitigated oxidative stress markers such as H_2_O_2_ and MDA, despite only modest effects on biomass production within the four-week study period. The improved metabolic responses and reduced stress indicators point to potential long-term benefits of the chelated micronutrient supply that may manifest more clearly over extended growth durations. Speciation and incubation studies reinforced these physiological findings, revealing that Fe-EDTA is unstable at pH values above 6.5, where Fe is prone to displacement by competing cations, particularly Ca^2+^, leading to the formation of insoluble Fe compounds. In contrast, Fe-HBED demonstrated superior chemical stability, effectively maintaining soluble Fe and preventing the precipitation of Fe and P under all alkalinity levels tested. The stability of Fe-HBED also correlated with the improved solubility of Ca, likely through indirect mechanisms that suppress the nucleation of calcium salts.

Overall, this study highlights the crucial role of chelate stability in determining micronutrient availability under alkaline conditions and demonstrates Fe-HBED as the most effective Fe source among the tested chelates under alkaline conditions. The work provides practical insights for optimizing micronutrient management in hydroponic and alkaline soil systems, advocating for the use of highly stable chelates like Fe-HBED to ensure the sustained availability of Fe and P and to improve plant resilience against alkaline stress.

The outcomes of this study underline the potential application of stable chelates, such as Fe-HBED in developing nutrient management strategies tailored for alkaline environments. While hydroponic trials provided clear mechanistic insights, the translation of these findings to soil-based and field-scale systems remains a critical next step. Future research should therefore focus on evaluating Fe-HBED and other stable Fe sources under diverse agroecological conditions, assessing their agronomic efficiency, cost-effectiveness, and compatibility with existing fertilization practices. Moreover, integrating chelate-based Fe management with innovative approaches, such as nanoscale Fe carriers,^55^ Fe-containing nanoclay polymer composites,^56^ organic amendments, and site-specific nutrient delivery, may offer practical pathways to mitigate Fe deficiency, enhance crop resilience, and promote sustainable intensification in alkaline and calcareous soils.

## Supplementary Material



## Notes

Nutrients’ speciation tables showing chelated and precipitated nutrient fractions; nonfully chelated micronutrient containing solutions at 0, 5, and 15 mM NaHCO_3_; fully chelated micronutrient containing solutions at 0, 5, and 15 mM NaHCO_3_ (PDF)

## §

Nuclear Institute for Agriculture and Biology College (NIAB-C), Pakistan Institute of Applied and Engineering Sciences, 38000 Faisalabad, Pakistan

## Abbreviations

Fe-EDTA: iron­(3^+^) ethylenediaminetetraacetic acid complex
Fe-DTPA: iron­(3^+^) diethylenetriaminepentaacetic acid complex
Fe-HBED: iron­(3^+^) *N*,*N*′-bis­(2-hydroxybenzyl)-ethylenediamine-*N*,*N*′-diacetic acid complex
AAS: atomic absorption spectrometer
ICP-OES: inductively coupled plasma optical emission spectrometer
IC: ion chromatography
MDA: malondialdehyde
XRD: X-ray diffraction
SEM-EDX: scanning electron microscopy with energy dispersive X-ray
