# Supplementary material for: Iron Solubility and Uptake in Fava Bean and Maize as a Function of Iron Chelates under Alkaline Hydroponic Conditions
Source: J Agric Food Chem. 2025 Oct 22;73(44):28100–16. doi: 10.1021/acs.jafc.5c08914 (PMC12593383; doi:10.1021/acs.jafc.5c08914)
Supplement: Supplementary file 1 [file jf5c08914_si_001.pdf]

# Supporting Information

# Iron solubility and uptake in faba bean and maize as a function of iron chelates under alkaline hydroponic conditions

Muhammad Faizan Ilyas[1], Muhammad Imran[2], Asif Naeem[1,3], Arjen M. Reichwein[2], and Karl Hermann Mühling[1*]

[1]Institute of Plant Nutrition and Soil Science, Kiel University, Hermann-Rodewald-Strasse 2, 24118 Kiel, Germany

[2]Nouryon Functional Chemicals, B.V., Zutphenseweg 10, 7418 AJ Deventer, The Netherlands

[3]Present address: Nuclear Institute for Agriculture and Biology College (NIAB-C), Pakistan Institute of Applied and Engineering Sciences, 38000 Faisalabad, Pakistan

*Corresponding author: Prof. Dr. Karl H. Mühling

E-mail: khmuehling@plantnutrition.uni-kiel.de

Phone: +49 431 880 3189

Table S1. Nutrients' speciation for non-fully chelated micronutrients containing solution at 0 mM $NaHCO_3$.

| pH | $Cu_{chel}$ (%) | $Fe_{chel}$ (%) | $Fe_{prec}$ (%) | $Mn_{chel}$ (%) | $Zn_{chel}$ (%) | $Ca_{chel}$ (%) | $Ca_{prec}$ (%) | $Mg_{chel}$ (%) | $Mg_{prec}$ (%) | $P_{prec}$ (%) |
|---|---|---|---|---|---|---|---|---|---|---|
| 4.0 | 58.2 | 99.9 | 0.1 | 0.0 | 0.7 | 0.0 | 0.0 | 0.0 | 0.0 | 0.1 |
| 5.0 | 99.1 | 99.7 | 0.2 | 0.2 | 37.4 | 0.0 | 0.0 | 0.0 | 0.0 | 0.2 |
| 6.0 | 100.0 | 99.4 | 0.6 | 11.3 | 98.1 | 0.0 | 0.0 | 0.0 | 0.0 | 0.6 |
| 7.0 | 100.0 | 77.6 | 22.4 | 96.1 | 100.0 | 1.3 | 6.7 | 0.0 | 0.0 | 73.7 |
| 8.0 | 100.0 | 1.1 | 98.9 | 99.3 | 100.0 | 5.9 | 14.5 | 0.1 | 0.0 | 97.9 |
| 9.0 | 100.0 | 0.8 | 99.3 | 100.0 | 100.0 | 5.2 | 93.7 | 4.9 | 32.0 | 0.0 |
| 10.0 | 100.0 | 3.1 | 97.0 | 100.0 | 100.0 | 5.1 | 94.8 | 4.8 | 88.1 | 0.0 |

All percentages are calculated based on the total concentration of nutrients present.

Table S2. Nutrients' speciation for non-fully chelated micronutrients containing solution at 5 mM $NaHCO_3$.

| pH | $Cu_{chel}$ (%) | $Fe_{chel}$ (%) | $Fe_{prec}$ (%) | $Mn_{chel}$ (%) | $Zn_{chel}$ (%) | $Ca_{chel}$ (%) | $Ca_{prec}$ (%) | $Mg_{chel}$ (%) | $Mg_{prec}$ (%) | $P_{prec}$ (%) |
|---|---|---|---|---|---|---|---|---|---|---|
| 4.0 | 57.8 | 99.9 | 0.1 | 0.0 | 0.7 | 0.0 | 0.0 | 0.0 | 0.0 | 0.1 |
| 5.0 | 99.1 | 99.7 | 0.2 | 0.1 | 37.2 | 0.0 | 0.0 | 0.0 | 0.0 | 0.2 |
| 6.0 | 100.0 | 99.4 | 0.6 | 11.2 | 98.0 | 0.0 | 0.0 | 0.0 | 0.0 | 0.6 |
| 7.0 | 100.0 | 77.7 | 22.3 | 96.1 | 100.0 | 1.3 | 6.5 | 0.0 | 0.0 | 71.9 |
| 8.0 | 100.0 | 1.1 | 98.9 | 99.2 | 100.0 | 5.9 | 12.0 | 0.1 | 0.0 | 97.8 |
| 9.0 | 100.0 | 0.8 | 99.3 | 100.0 | 100.0 | 5.2 | 93.7 | 4.9 | 30.5 | 0.0 |
| 10.0 | 100.0 | 3.1 | 96.9 | 100.0 | 100.0 | 5.1 | 94.8 | 4.8 | 88.1 | 0.0 |

All percentages are calculated based on the total concentration of nutrients present.

Table S3. Nutrients' speciation for non-fully chelated micronutrients containing solution at 15 mM $NaHCO_3$.

| pH | $Cu_{chel}$ (%) | $Fe_{chel}$ (%) | $Fe_{prec}$ (%) | $Mn_{chel}$ (%) | $Zn_{chel}$ (%) | $Ca_{chel}$ (%) | $Ca_{prec}$ (%) | $Mg_{chel}$ (%) | $Mg_{prec}$ (%) | $P_{prec}$ (%) |
|---|---|---|---|---|---|---|---|---|---|---|
| 4.0 | 57.2 | 99.9 | 0.1 | 0.0 | 0.7 | 0.0 | 0.0 | 0.0 | 0.0 | 0.1 |
| 5.0 | 99.1 | 99.8 | 0.2 | 0.1 | 36.7 | 0.0 | 0.0 | 0.0 | 0.0 | 0.2 |
| 6.0 | 100.0 | 99.4 | 0.6 | 10.9 | 98.0 | 0.0 | 0.0 | 0.0 | 0.0 | 0.6 |
| 7.0 | 100.0 | 77.9 | 22.1 | 96.0 | 100.0 | 1.3 | 6.2 | 0.0 | 0.0 | 68.5 |
| 8.0 | 100.0 | 1.1 | 98.9 | 99.2 | 100.0 | 5.9 | 8.9 | 0.1 | 0.0 | 97.6 |
| 9.0 | 100.0 | 0.8 | 99.2 | 100.0 | 100.0 | 5.2 | 93.7 | 4.9 | 27.7 | 0.0 |
| 10.0 | 100.0 | 3.1 | 96.9 | 100.0 | 100.0 | 5.1 | 94.8 | 4.8 | 88.1 | 0.0 |

All percentages are calculated based on the total concentration of nutrients present.

Table S4. Nutrients' speciation for fully chelated micronutrients containing solution at 0 mM $NaHCO_3$.

| pH | $Cu_{chel}$ (%) | $Fe_{chel}$ (%) | $Fe_{prec}$ (%) | $Mn_{chel}$ (%) | $Zn_{chel}$ (%) | $Ca_{chel}$ (%) | $Ca_{prec}$ (%) | $Mg_{chel}$ (%) | $Mg_{prec}$ (%) | $P_{prec}$ (%) |
|---|---|---|---|---|---|---|---|---|---|---|
| 4.0 | 100.0 | 100.0 | 0.0 | 25.5 | 99.2 | 0.0 | 0.0 | 0.0 | 0.0 | 0.0 |
| 5.0 | 100.0 | 100.0 | 0.0 | 39.2 | 99.6 | 0.0 | 0.0 | 0.0 | 0.0 | 0.0 |
| 6.0 | 100.0 | 100.0 | 0.0 | 39.6 | 99.6 | 0.0 | 0.0 | 0.0 | 0.0 | 0.0 |
| 7.0 | 100.0 | 78.7 | 21.3 | 96.1 | 100.0 | 1.3 | 6.7 | 0.0 | 0.0 | 73.7 |
| 8.0 | 100.0 | 1.1 | 98.9 | 99.3 | 100.0 | 6.0 | 14.4 | 0.1 | 0.0 | 97.9 |
| 9.0 | 100.0 | 0.8 | 99.2 | 100.0 | 100.0 | 5.3 | 93.7 | 5.0 | 32.0 | 0.0 |
| 10.0 | 100.0 | 3.1 | 96.9 | 100.0 | 100.0 | 5.1 | 94.7 | 4.9 | 88.0 | 0.0 |

All percentages are calculated based on the total concentration of nutrients present.

Table S5. Nutrients' speciation for fully chelated micronutrients containing solution at 5 mM $NaHCO_3$.

| pH | $Cu_{chel}$ (%) | $Fe_{chel}$ (%) | $Fe_{prec}$ (%) | $Mn_{chel}$ (%) | $Zn_{chel}$ (%) | $Ca_{chel}$ (%) | $Ca_{prec}$ (%) | $Mg_{chel}$ (%) | $Mg_{prec}$ (%) | $P_{prec}$ (%) |
|---|---|---|---|---|---|---|---|---|---|---|
| 4.0 | 100.0 | 100.0 | 0.0 | 25.2 | 99.2 | 0.0 | 0.0 | 0.0 | 0.0 | 0.0 |
| 5.0 | 100.0 | 100.0 | 0.0 | 39.1 | 99.6 | 0.0 | 0.0 | 0.0 | 0.0 | 0.0 |
| 6.0 | 100.0 | 100.0 | 0.0 | 39.5 | 99.6 | 0.0 | 0.0 | 0.0 | 0.0 | 0.0 |
| 7.0 | 100.0 | 78.8 | 21.2 | 96.1 | 100.0 | 1.3 | 6.5 | 0.0 | 0.0 | 71.9 |
| 8.0 | 100.0 | 1.1 | 98.9 | 99.3 | 100.0 | 6.0 | 11.9 | 0.1 | 0.0 | 97.8 |
| 9.0 | 100.0 | 0.8 | 99.2 | 100.0 | 100.0 | 5.3 | 93.6 | 5.0 | 30.4 | 0.0 |
| 10.0 | 100.0 | 3.1 | 96.9 | 100.0 | 100.0 | 5.1 | 94.7 | 4.9 | 88.0 | 0.0 |

All percentages are calculated based on the total concentration of nutrients present.

Table S6. Nutrients' speciation for fully chelated micronutrients containing solution at 15 mM $NaHCO_3$.

| pH | $Cu_{chel}$ (%) | $Fe_{chel}$ (%) | $Fe_{prec}$ (%) | $Mn_{chel}$ (%) | $Zn_{chel}$ (%) | $Ca_{chel}$ (%) | $Ca_{prec}$ (%) | $Mg_{chel}$ (%) | $Mg_{prec}$ (%) | $P_{prec}$ (%) |
|---|---|---|---|---|---|---|---|---|---|---|
| 4.0 | 100.0 | 100.0 | 0.0 | 24.6 | 99.2 | 0.0 | 0.0 | 0.0 | 0.0 | 0.0 |
| 5.0 | 100.0 | 100.0 | 0.0 | 39.1 | 99.6 | 0.0 | 0.0 | 0.0 | 0.0 | 0.0 |
| 6.0 | 100.0 | 100.0 | 0.0 | 39.5 | 99.6 | 0.0 | 0.0 | 0.0 | 0.0 | 0.0 |
| 7.0 | 100.0 | 79.0 | 21.0 | 96.1 | 100.0 | 1.3 | 6.2 | 0.0 | 0.0 | 68.5 |
| 8.0 | 100.0 | 1.1 | 98.9 | 99.2 | 100.0 | 6.0 | 8.9 | 0.1 | 0.0 | 97.6 |
| 9.0 | 100.0 | 0.8 | 99.2 | 100.0 | 100.0 | 5.3 | 93.6 | 5.0 | 27.6 | 0.0 |
| 10.0 | 100.0 | 3.1 | 96.9 | 100.0 | 100.0 | 5.1 | 94.7 | 4.9 | 88.0 | 0.0 |

All percentages are calculated based on the total concentration of nutrients present.
